# Small-Maturity Asymptotics for the At-The-Money Implied Volatility Slope in Lévy Models

**DOI:** 10.1080/1350486X.2016.1197041

**Published:** 2016-06-30

**Authors:** Stefan Gerhold, I. Cetin Gülüm, Arpad Pinter

**Affiliations:** ^a^ Institute of Statistics and Mathematical Methods in Economics, TU Wien, Wiedner Hauptstrasse 8–10/E105-1, ViennaA-1040, Austria

**Keywords:** Implied volatility, Lévy process, digital option, asymptotics, Mellin transform

## Abstract

We consider the at-the-money (ATM) strike derivative of implied volatility as the maturity tends to zero. Our main results quantify the behaviour of the slope for infinite activity exponential Lévy models including a Brownian component. As auxiliary results, we obtain asymptotic expansions of short maturity ATM digital call options, using Mellin transform asymptotics. Finally, we discuss when the ATM slope is consistent with the steepness of the smile wings, as given by Lee’s moment formula.

## 1. Introduction

Recent years have seen an explosion of the literature on asymptotics of option prices and implied volatilities (see, e.g., Andersen and Lipton 2013; Friz et al. 2011 for further details). Such results are of practical relevance for fast model calibration, qualitative model assessment and parametrization design. The small-time behaviour of the *level* of implied volatility in Lévy models (and generalizations) has been investigated in great detail (Boyarchenko and Levendorski 2002; Figueroa-López and Forde 2012; Figueroa-López, Gong, and Houdré 2012, 2014; Roper 2008; Tankov 2011). We, on the other hand, focus on the at-the-money (ATM) *slope* of implied volatility, i.e., the strike derivative, and investigate its behaviour as maturity becomes small. For diffusion models, there typically exists a limiting smile as the maturity tends to zero, and the limit slope is just the slope of this limit smile (e.g., for the Heston model, this follows from Section 5 in Durrleman 2010b). Our focus is, however, on exponential Lévy models. There is no limit smile here that one could differentiate, as the implied volatility blows up off-the-money (Tankov 2011). In fact, this is a desirable feature, since in this way Lévy models are better suited to capture the steep short maturity smiles observed in the market. But it also implies that the limiting slope cannot be deduced directly from the behaviour of implied volatility itself, and requires a separate analysis. (Note that a limiting smile does exist if maturity and log-moneyness tend to zero jointly in an appropriate way (Mijatovi and Tankov 2012).)

It turns out that the presence of a Brownian component has a decisive influence: without it, the ATM slope explodes (under mild conditions). The blow-up is of order *T* ^–1/2^ for many models, but may also be slower (CGMY model with *Y* ∈ (1, 2), e.g.; see Example 10). Our main results are on Lévy models *with* a Brownian component, though. We provide a result (Corollary 6 in Section 5) that translates the asymptotic behaviour of the moment generating function to that of the ATM slope. When applied to concrete models, we see that the slope may converge to a finite limit (Normal Inverse Gaussian (NIG), Meixner, CGMY models) or explode at a rate slower than *T* ^–1/2^ (generalized tempered stable model; this kind of behaviour seems to be the most realistic one, see Bayer, Friz, and Gatheral 2016). Note that several studies (Aït-Sahalia 2002, Aït-Sahalia and Jacod, 2010; Carr and Wu 2003) highlight the importance of a Brownian component when fitting to historical data or option prices. In particular, in many pure jump Lévy models, ATM implied volatility converges to zero as *T* ↓ 0 (see Proposition 5 in Tankov 2011 for a precise statement), which seems undesirable.

From a practical point of view, the asymptotic slope is a useful ingredient for model calibration; e.g., if the market slope is negative, then a simple constraint on the model parameters forces the (asymptotic) model slope to be negative, too. Our numerical tests show that the sign of the slope is reliably identified by a first-order asymptotic approximation, even if the maturity is not short at all. With our formulas, the asymptotic slope (and, of course, its sign) can be easily determined from the model parameters. For instance, the slope of the NIG model is positive if and only if the skewness parameter satisfies $\beta >-\frac{1}{2}$.

To obtain these results, we investigate the asymptotics of ATM digital calls; their relation to the implied volatility slope is well known. While, for Lévy processes *X*, the small-time behaviour of the transition probabilities $P[X_{T}\geq x]$ (in finance terms, digital call prices) has been well studied for *x* ≠ *X*_0_ (see, e.g., Figueroa-López and Houdré 2009 and the references therein), not so much is known for *x* = *X*_0_. Still, first-order asymptotics of $P[X_{T}\geq X_{0}]$ are available, and this suffices if there is no Brownian component. If the Lévy process has a Brownian component, then it is well known that $\lim_{T\to 0}P[X_{T}\geq X_{0}]=\frac{1}{2}$. In this case, it turns out that the second-order term of $P[X_{T}\geq X_{0}]$ is required to obtain slope asymptotics. For this, we use a novel approach involving the Mellin transform (w.r.t. time) of the transition probability (Sections 4 and 5). We believe that this method is of wide applicability to other problems involving time asymptotics of Lévy processes, and hope to elaborate on it in future work.

Finally, we consider the question whether a positive ATM slope requires the right smile wing to be the steeper one, and vice versa. Wing steepness refers to large-strike asymptotics here. It turns out that this is indeed the case for several of the infinite activity models we consider. This results in a qualitative limitation on the smile shape that these models can produce.

One of the few other works dealing with small-time Lévy slope asymptotics is the comprehensive recent paper by Andersen and Lipton (2013). Besides many other problems on various models and asymptotic regimes, they study the small-maturity ATM digital price and volatility slope for the tempered stable model (Propositions 8.4 and 8.5 in Andersen and Lipton 2013). This includes the CGMY model as a special case (see Example 10 for details). Their proof method is entirely different from ours, exploiting the explicit form of the characteristic function of the tempered stable model. Using mainly the dominated convergence theorem, they also analyse the convexity. We, on the other hand, assume a certain asymptotic behaviour of the characteristic function, and use its explicit expression only when calculating concrete examples. Our approach covers, e.g., the ATM slope of the generalized tempered stable, NIG and Meixner models without additional effort.

The recent preprint (Figueroa-López and Ólafsson 2015) is also closely related to our work. There, the Brownian component is generalized to stochastic volatility. On the other hand, the assumptions on the Lévy measure exclude, e.g., the NIG and Meixner models. Section 6 has additional comments on how our results compare to those of Andersen and Lipton (2013) and Figueroa-López and Ólafsson (2015). Alòs, León, and Vives (2007) also study the small time implied volatility slope under stochastic volatility and jumps, but the latter are assumed to have finite activity, which is not our focus. Results on the *large* time slope can be found in Forde, Jacquier, and Figueroa-López (2011); see also Gatheral (2006, p. 63f).

## 2. Digital Call Prices

We denote the underlying by $S=e^{X}$, normalized to $S_{0}=1$, and the pricing measure by ℙ. W.l.o.g. the interest rate is set to zero, and so *S* is a ℙ-martingale. Suppose that the log-underlying $X=(X_{t}{)}_{t\geq 0}$ is a Lévy process with characteristic triplet $\left(b,\sigma^{2},\nu \right)$ and $X_{0}=0$. The moment generating function (mgf) of *X_T_* is

$$M(z,T)=E[e^{zX_{T}}]=\exp\left(T\psi (z)\right),$$

where

(2.1) $$\psi (z)=\frac{1}{2}\sigma^{2}z^{2}+bz+\int_{R}(e^{zx}-1-zx)\nu (dx).$$

This representation is valid if the Lévy process has a finite first moment, which we of course assume, as even $S_{t}=e^{X_{t}}$ should be integrable. If, in addition, *X* has paths of finite variation, then $\int_{R}|x|\nu (dx)<\infty$, and

$$\psi (z)=\frac{1}{2}\sigma^{2}z^{2}+b_{0}z+\int_{R}(e^{zx}-1)\nu (dx),$$

where the drift $b_{0}$ is defined by

$$b_{0}=b-\int_{R}x\nu (dx).$$

The following theorem collects some results about the small-time behaviour of $P[X_{T}\geq 0]$. All of them are known, or easily obtained from known results. We are mainly interested in the case where $S=e^{X}$ is a martingale, and so $P[X_{T}\geq 0]$ has the interpretation of an ATM digital call price. Still, we mention that this assumption is not necessary for parts (i)–(iv). In part (iv), the following condition from Rosenbaum and Tankov (2011) is used:

$$(H-\alpha )\;The\;Le{\;}^{\prime }vy\;measure\;\nu \;has\;a\;density\;g(x)/\;|x|{\;}^{1+\alpha },\;where\;g\;is\;a\;non-negative$$

measurable function admitting left and right limits at zero:

$$c_{+}:=\underset{x↓0}{\lim}g(x),c_{-}:=\underset{x↑0}{\lim}g(x),withc_{+}+c_{-}>0.$$

Theorem 1. *Let X be a Lévy process with characteristic triplet* $\left(b,\sigma^{2},\nu \right)$ *and X*_0_ = 0.

- If *X has finite variation, and b*_0_ ≠ 0, *then*
  $$\underset{T↓0}{\lim}P[X_{T}\geq 0]=\{\begin{array}{cc} 1, & b_{0}>0\\ 0, & b_{0}<0. \end{array}$$
- If *σ* > 0, *then* $\lim_{T↓0}P[X_{T}\geq 0]=\frac{1}{2}$.
- If *X is a Lévy jump diffusion, i.e., it has finite activity jumps and σ* > 0, *then*
  $$P[X_{T}\geq 0]=\frac{1}{2}+\frac{b_{0}}{\sigma \sqrt{2\pi }}\sqrt{T}+O(T),T↓0.$$
- *Suppose that σ* = 0 *and that* (H-α) *holds for some* α∈[1,2). If α=1, *we additionally assume* $c_{-}=c_{+}=:c$ *and* $\int_{0}^{1}x^{-1}|\;g(x)-g(-x)|dx<\infty$. *Then*
  $$\underset{T↓0}{\lim}P[X_{T}\geq 0]=\{\begin{array}{cc} \frac{1}{2}+\frac{1}{\pi }\arctan\frac{b^{*}}{\pi c} & if\alpha =1,\\ \frac{1}{2}+\frac{\alpha }{\pi }\arctan(\beta \tan(\frac{\alpha \pi }{2})) & if\alpha \neq 1, \end{array}$$
*where* $b^{*}=b-\int_{0}^{\infty }(g(x)-g(-x))/xdx$ *and* $\beta =(c_{+}-c_{-})/(c_{+}+c_{-}).$
- *If e^X^ is a martingale and the Lévy measure satisfies* $\nu (dx)=e^{-x/2}\nu_{0}(dx)$, *where v*_0_ *is a symmetric measure, then*
  $$P[X_{T}\geq 0]=\Phi (-\sigma_{imp}(1,T)\sqrt{T}/2),$$
*where* Φ *denotes the standard Gaussian cdf*.

*Proof*. (i) We have $P[X_{T}\geq 0]=P[T^{-1}X_{T}\geq 0]$, but T^–1^ *X_T_* converges a.s. to *b*_0_, by Theorem 43.20 in (Sato 1999).

(ii) If σ>0, then $T^{-1/2}X_{T}$ converges in distribution to a centred Gaussian random variable with variance *σ* ^2^ (see Sato 1999). For further central limit theorem-type results in this vein, see Doney and Maller (2002) and Gerhold et al. (2015).

(iii) Conditioning on the first jump time *τ*, which has an exponential distribution, we find

(2.2) $$\begin{array}{rl} P[X_{T}\geq 0] & =P[X_{T}\geq 0|\tau \leq T]\cdot P[\tau \leq T]+P[X_{T}\geq 0|\tau >T]\cdot P[\tau >T]\\ & =O(T)+P[\sigma W_{T}+b_{0}T\geq 0](1+O(T))\\ & =P[\sigma W_{T}+b_{0}T\geq 0]+O(T)\\ & =\Phi (b_{0}\sqrt{T}/\sigma )+O(T). \end{array}$$

Now apply the expansion

(2.3) $$\Phi (x)=\frac{1}{2}+\frac{x}{\sqrt{2\pi }}+O(x^{3}),x\to 0.$$

(iv) By Proposition 1 in Rosenbaum and Tankov (2011), the rescaled process $X_{t}^{\varepsilon ,\alpha }:=\varepsilon^{-1}X_{\varepsilon^{\alpha }t}$ converges in law to a strictly *α*-stable process $X_{t}^{*,\alpha }$ as *ε*↓0. Therefore,

$$\underset{T↓0}{\lim}P[X_{T}\geq 0]=\underset{\varepsilon ↓0}{\lim}P[\varepsilon^{-1}X_{\varepsilon^{\alpha }}\geq 0]=P[X_{1}^{*,\alpha }\geq 0],$$

and it suffices to evaluate the latter probability. For α=1, $X_{1}^{*,1}$ has a Cauchy distribution with characteristic exponent

$$\log E[\exp(iuX_{1}^{*,1})]=ib^{*}u-\pi c|u|,$$

hence $P[X_{1}^{*,1}\geq 0]=\frac{1}{\pi }\arctan\frac{b^{*}}{\pi c}$. (Our b* is denoted γ* in Rosenbaum and Tankov (2011).)

If 1 < *α* < 2, then $X_{1}^{*,\alpha }$ has a strictly stable distribution with characteristic exponent

$$\log E[\exp(iuX_{1}^{*,\alpha })]=-|du{|}^{\alpha }\left(1-i\beta sgn(u)\tan\left(\frac{\alpha \pi }{2}\right)\right),$$

where

$$d_{\pm }^{\alpha }=-\Gamma (-\alpha )\cos\left(\frac{\alpha \pi }{2}\right)c_{\pm }\geq 0,d^{\alpha }=d_{+}^{\alpha }+d_{-}^{\alpha },\beta =\frac{d_{+}^{\alpha }-d_{-}^{\alpha }}{d^{\alpha }}\in (-1,1).$$

The desired expression for $P[X_{1}^{*,\alpha }\geq 0]$ then follows from Davydov and Ibragimov (1971). See Figueroa-López and Forde (2012) for further related references.

(v) Under this assumption, the market model is symmetric in the sense of Fajardo (2015) and Fajardo and Mordecki (2006). The statement is Theorem 3.1 in Fajardo (2015).

The variance gamma model and the CGMY model with 0 < *Y* < 1 are examples of finite variation models (of course, only when *σ* = 0), and so part (i) of Theorem 1 is applicable. Part (iii) is applicable, clearly, to the well-known jump diffusion models by Merton and Kou. In Section 6, we will discuss two examples for part (iv) (NIG and Meixner).

## 3. Implied Volatility Slope and Digital Options with Small Maturity

The (Black–Scholes) implied volatility is the volatility that makes the Black–Scholes call price equal the call price with underlying *S*:

$$C_{BS}(K,T,\sigma_{imp}(K,T))=C(K,T):=E[(S_{T}-K{)}^{+}].$$

Since no explicit expression is known for $\sigma_{imp}(K,T)$ (see Gerhold (2013)), many authors have investigated approximations (see, e.g., the references in the introduction). The following relation between implied volatility slope and digital calls is well known (Gatheral 2006); we give a proof for completeness. (Note that absolute continuity of *S_T_* holds in all Lévy models of interest, see Theorem 27.4 in Sato (1999), and will be assumed throughout.)

Lemma 2. *Suppose that the law of S_T_ is absolutely continuous for each T *> 0, *and that*

(3.1) $$\underset{T↓0}{\lim}C(K,T)=(S_{0}-K{)}^{+},K>0.$$

*Then, for T* ↓ 0,

(3.2) $$\partial_{K}\sigma_{\text{imp}}(K,T){|}_{K=1}\sim \sqrt{\frac{2\pi }{T}}\left(\frac{1}{2}-\mathbb{P}[S_{T}\geq 1]-\frac{\sigma_{\text{imp}}(1,T)\sqrt{T}}{2\sqrt{2\pi }}+O({\left(\sigma_{\text{imp}}(1,T)\sqrt{T}\right)}^{2})\right).$$

*Proof*. By the implicit function theorem, the implied volatility slope has the representation

$$\partial_{K}\sigma_{imp}(K,T)=\frac{\partial_{K}C(K,T)-\partial_{K}C_{BS}(K,T,\sigma_{imp}(K,T))}{\partial_{\sigma }C_{BS}(K,T,\sigma_{imp}(K,T))}.$$

Since the law of *S_T_* is absolutely continuous, the call price *C*(*K,T*) is continuously differentiable w.r.t. *K*, and $\partial_{K}C(K,T)=-P[S_{T}\geq K]$. Inserting the explicit formulas for the Black–Scholes Vega and digital price, and specializing to the ATM case *K* = *S*_0_ = 1, we get

$$\partial_{K}\sigma_{imp}(K,T){|}_{K=1}=\frac{\Phi (-\sigma_{imp}(1,T)\sqrt{T}/2)-P[S_{T}\geq 1]}{\sqrt{T}\varphi (\sigma_{imp}(1,T)\sqrt{T}/2)},$$

where Φ and *φ* denote the standard Gaussian cdf and density, respectively. By Proposition 4.1 in Roper and Rutkowski (2009), our assumption (Equation (3.1)) implies that the annualized implied volatility $\sigma_{imp}(1,T)\sqrt{T}$ tends to zero as *T* ↓ 0. (The second assumption used in Roper and Rutkowski (2009) are the no-arbitrage bounds $(S_{0}-K{)}^{+}\leq C(K,T)\leq S_{0}$, for K,T>0, but these are satisfied here because our call prices are generated by the martingale *S*.) Using Equation (2.3) and $\varphi (x)=\frac{1}{\sqrt{2\pi }}+O(x^{2})$, we thus obtain Equation (3.2).

The asymptotic relation (Equation (3.2)) is, of course, consistent with the small-moneyness expansion presented in De Leo et al. (2012), where $\sqrt{2\pi /T}\left(\frac{1}{2}-P[S_{T}\geq K]\right)$ appears as second-order term (i.e., first derivative) of implied volatility.

Lemma 2 shows that, in order to obtain first-order asymptotics for the ATM slope, we need first-order asymptotics for the ATM digital call price $P[S_{T}\geq 1]$. (Recall that *S*_0_ = 1.) For models where $\lim_{T↓0}P[S_{T}\geq 1]=\frac{1}{2}$, we need the second-order term of the digital call as well, and the first-order term of $\sigma_{imp}(1,T)\sqrt{T}$. The limiting value 1/2 for the ATM digital call is typical for diffusion models (see Gerhold et al. 2015), and Lévy processes that contain a Brownian motion. For infinite activity models without diffusion component, $P[S_{T}\geq 1]$ may converge to 1/2 as well (e.g., in the CGMY model with Y∈(1,2)), but other limiting values are also possible. See the examples in Section 6.

From part (i) of Theorem 1 and Lemma 2, we can immediately conclude the following result. Note that we assume throughout that *X* is such that $S=e^{X}$ is a martingale with *S*_0_ = 1.

Proposition 3. *Suppose that the Lévy process X has finite variation (and thus, necessarily, that* σ=0 *), and that* $b_{0}\neq 0$. *Then the ATM implied volatility slope satisfies*

$$\partial_{K}\sigma_{imp}(K,T){|}_{K=1}∼-\sqrt{\pi /2}sgn(b_{0})\cdot T^{-1/2},T↓0.$$

Note that *T* ^–1/2^ is the fastest possible growth order for the slope, in any model (see Lee 2005).

If *X* is a Lévy jump diffusion with σ>0, then by part (iii) of Theorem 1 (Equation (3.2)) and the fact that $\sigma_{imp}\to \sigma$ (implied volatility converges to spot volatility), we obtain the finite limit

(3.3) $$\underset{T↓0}{\lim}\partial_{K}\sigma_{imp}(K,T){|}_{K=1}=-\frac{b_{0}}{\sigma }-\frac{\sigma }{2}.$$

(It is understood that the substitution K=1 is to be performed before the limit *T* ↓ 0.) Notice that the expression on the right-hand side of Equation (3.3) does depend on the jump parameters, because the drift *b*_0_, fixed by the condition $E[\exp(X_{1})]=1$, depends on them. Moreover, Equation (3.3) is consistent with the formal calculation of the variance slope

$$\underset{T↓0}{\lim}\partial_{K}\sigma_{imp}^{2}(K,T){|}_{K=1}=-2b_{0}-\sigma^{2}$$

in Gatheral (2006, p. 61f). In fact Equation (3.3) is well known for jump diffusions, see also Alòs, León, and Vives (2007) and Yan (2011).

## 4. General Remarks on Mellin Transform Asymptotics

As mentioned after Lemma 2, we need the second-order term for the ATM digital call if we want to find the limiting slope in Lévy models with a Brownian component. While this is easy for finite activity models (see the end of Section 3), it is more difficult in the case of infinite activity jumps. We will find this second-order term using Mellin transform asymptotics. For further details and references on this technique, see, e.g., Flajolet, Gourdon, and Dumas (1995). The Mellin transform of a function *H*, locally integrable on (0, ∞), is defined by

$$(MH)(s)=\int_{0}^{\infty }T^{s-1}H(T)dT.$$

Under appropriate growth conditions on *H* at zero and infinity, this integral defines an analytic function in an open vertical strip of the complex plane. The function *H* can be recovered from its transform by Mellin inversion (see formula (7) in Flajolet, Gourdon, and Dumas 1995):

(4.1) $$H(T)=\frac{1}{2\pi i}\int_{\kappa -i\infty }^{\kappa +i\infty }(MH)(s)T^{-s}ds,$$

where κ is a real number in the strip of analyticity of ℳ*H*. For the validity of Equation (4.1), it suffices that *H* is continuous and that y↦(MH)(κ+iy) is integrable. Denote by $s_{0}\in R$ the real part of the left boundary of the strip of analyticity. A typical situation in applications is that ℳ*H* has a pole at *s*_0_, and admits a meromorphic extension to a left half-plane, with further poles at $s_{0}>s_{1}>s_{2}>\ldots$ Suppose also that the meromorphic continuation satisfies growth estimates at ±*i∞* which allow to shift the integration path in Equation (4.1) to the left. We then collect the contribution of each pole by the residue theorem, and arrive at an expansion (see formula (8) in Flajolet, Gourdon, and Dumas 1995)

$$H(T)=Res_{s=s_{0}}(MH)(s)T^{-s}+Res_{s=s_{1}}(MH)(s)T^{-s}+\ldots$$

Thus, the basic principle is that singularities *s_i_* of the transform are mapped to terms $T^{-s_{i}}$ in the asymptotic expansion of *H* at zero. Simple poles of ℳ*H* yield powers of *T*, whereas double poles produce an additional logarithmic factor log*T*, as seen from the expansion $T^{-s}=T^{-s_{i}}(1-(\log T)(s-s_{i})+O((s-s_{i}{)}^{2}))$.

## 5. Main Results: Digital Call Prices and Slope Asymptotics

The mgf M(z,T) of $X_{T}$ is analytic in a strip $z_{-}<Re(z)<z_{+}$, given by the critical moments

(5.1) $$z_{+}=\sup\{z\in R:E[e^{zX_{T}}]<\infty \}$$

and

(5.2) $$z_{-}=\inf\{z\in R:E[e^{zX_{T}}]<\infty \}.$$

Since *X* is a Lévy process, the critical moments do not depend on *T*. We will obtain asymptotic information on the transition probabilities (i.e., digital call prices) from the Fourier representation (Lee 2004b)

(5.3) $$\begin{array}{rl} P[S_{T}\geq 1] & =P[X_{T}\geq 0]\\ & =\frac{1}{2i\pi }\int_{a-i\infty }^{a+i\infty }\frac{M(z,T)}{z}dz\\ & =\frac{1}{\pi }Re\int_{0}^{\infty }\frac{M(a+iy,T)}{a+iy}dy, \end{array}$$

where the real part of the vertical integration contour satisfies $a\in (0,1)\subseteq (z_{-},z_{+})$, and convergence of the integral is assumed throughout. We are going to analyse the asymptotic behaviour of this integral, for T↓0, by computing its Mellin transform. Asymptotics of the probability (digital price) $P[X_{T}\geq 0]$ are then evident from Equation (5.3). The linearity of log *M* as a function of *T* enables us to evaluate the Mellin transform in semi-explicit form.

Lemma 4. *Suppose that* $S=e^{X}$ *is a martingale, and that* σ>0. *Then, for any* a∈(0,1), *the Mellin transform of the function*

(5.4) $$H(T):=\int_{0}^{\infty }\frac{e^{T\psi (a+iy)}}{a+iy}dy,T>0,$$

is given by

(5.5) $$(MH)(s)=\Gamma (s)F(s),0<Re(s)<\frac{1}{2},$$

where

(5.6) $$F(s)=\int_{0}^{\infty }\frac{{\left(-\psi (a+iy)\right)}^{-s}}{a+iy}dy,0<Re(s)<\frac{1}{2}.$$

*Moreover*, |(MH)(s)| *decays exponentially, if* $Re(s)\in (0,\frac{1}{2})$ *is fixed and* |Im(s)|→∞.

See the Appendix for the proof of Lemma 4. With the Mellin transform in hand, we now proceed to convert an expansion of the mgf at *i*∞ to an expansion of $P[X_{T}\geq 0]$ for T↓0. The following result covers, e.g., the NIG and Meixner models, and the generalized tempered stable model, all with σ>0. See Section 6 for details.

Theorem 5. *Suppose that* $S=e^{X}$ *is a martingale, and that* σ>0. *Assume further that there are constants* a∈(0,1), c∈C, ν∈[1,2) *and* ε>0 *such that the Laplace exponent satisfies*

(5.7) $$\psi (z)=\frac{1}{2}\sigma^{2}z^{2}+cz^{\nu }+O(z^{\nu -\varepsilon }),Re(z)=a,Im(z)\to \infty .$$

*Then the ATM digital call price satisfies*

(5.8) $$P[X_{T}\geq 0]=\frac{1}{2}+C_{\tilde{\nu }}T^{\tilde{\nu }}+o(T^{\tilde{\nu }}),T↓0,$$

*where* $C_{\tilde{\nu }}=\frac{\tilde{\nu }}{2\pi }{\left(\frac{1}{2}\sigma^{2}\right)}^{\tilde{\nu }-1}Im(e^{-i\pi \tilde{\nu }}c)\Gamma (-\tilde{\nu })$ *with* $\tilde{\nu }=(2-\nu )/2\in (0,\frac{1}{2}]$. For ν=1, *this simplifies to*

$$P[X_{T}\geq 0]=\frac{1}{2}+\frac{Re(c)}{\sigma \sqrt{2\pi }}\sqrt{T}+o(\sqrt{T}),T↓0.$$

Together with Lemma 2, this theorem implies the following corollary, which is our main result on the implied volatility slope as T↓0.

Corollary 6. *Under the assumptions of Theorem 5, the ATM implied volatility slope behaves as follows:*

(i) If ν=1, then

$$\underset{T↓0}{\lim}\partial_{K}\sigma_{imp}(K,T){|}_{K=1}=-\frac{Re(c)}{\sigma }-\frac{\sigma }{2},$$

with *c* from Equation (5.7).

(ii) If 1<ν<2 and $C_{\tilde{\nu }}\;\neq \;0$, then

$$\partial_{K}\sigma_{imp}(K,T){|}_{K=1}∼-\sqrt{2\pi }C_{\tilde{\nu }}T^{\tilde{\nu }-1/2},T↓0.$$

*Proof of Theorem*. From Equations (5.3) and (5.4), we know that

(5.9) $$P[X_{T}\geq 0]=\frac{1}{\pi }ReH(T).$$

We now express *H*(*T*) by the Mellin inversion formula (Equation (4.1)), with $\kappa \in (0,\frac{1}{2})$. This is justified by Lemma 4, which yields the exponential decay of the transform *ℳH* along vertical rays. (Continuity of *H*, which is also needed for the inverse transform, is clear.) Therefore, we have

(5.10) $$H(T)=\frac{1}{2\pi i}\int_{1/4-i\infty }^{1/4+i\infty }\Gamma (s)F(s)T^{-s}ds,T\geq 0.$$

As outlined in Section 4, we now show that Γ(s)F(s) has a meromorphic continuation, then shift the integration path in Equation (5.10) to the left, and collect residues. It is well known that Γ is meromorphic with poles at the non-positive integers, so it suffices to discuss the continuation of *F*, defined in Equation (5.6). As in the proof of Lemma 4, we put h(y):=−ψ(a+iy), y≥0. To prove exponential decay of the desired meromorphic continuation, it is convenient to split the integral:

(5.11) $$\begin{array}{rl} F(s) & =\int_{0}^{y_{0}}\frac{h{\left(y\right)}^{-s}}{a+iy}dy+\int_{y_{0}}^{\infty }\frac{h{\left(y\right)}^{-s}}{a+iy}dy\\ & =:A_{0}(s)+\tilde{F}(s),0<Re(s)<\frac{1}{2}. \end{array}$$

The constant $y_{0}\geq 0$ will be specified later. It is easy to see that *A*_0_ is analytic in the half-plane $Re(s)<\frac{1}{2}$, and so $\tilde{F}$ captures all poles of *F* in that half-plane. By Equation (5.7), the function *h* has the expansion (with a possibly decreased ε, to be precise)

(5.12) $$h(y)=\frac{1}{2}\sigma^{2}y^{2}+\tilde{c}y^{\nu }+O(y^{\nu -\varepsilon }),y\to \infty ,$$

where

$$\tilde{c}:=\{\begin{array}{cc} -ci^{\nu } & \nu >1\\ -(c+\sigma^{2}a)i & \nu =1. \end{array}$$

The reason why *F* (or $\tilde{F}$) is not analytic at *s* = 0 is that the second integral in Equation (5.11) fails to converge for *y* large. We thus subtract the following convergence-inducing integral from $\tilde{F}$:

(5.13) $$\begin{array}{rl} {\tilde{G}}_{1}(s): & =\int_{y_{0}}^{\infty }\frac{{\left(\frac{1}{2}\sigma^{2}y^{2}\right)}^{-s}}{a+iy}dy\\ & =-\pi i(\frac{1}{2}a^{2}\sigma^{2}{)}^{-s}\frac{e^{i\pi s}}{\sin2\pi s}-\int_{0}^{y_{0}}\frac{{\left(\frac{1}{2}\sigma^{2}y^{2}\right)}^{-s}}{a+iy}dy \end{array}$$

$$=:G_{1}(s)+A_{1}(s).$$

Note that *G*_1_ is meromorphic, and that *A*_1_ is analytic for $Re(s)<\frac{1}{2}$. From the expansion

(5.14) $$h(y{)}^{-s}=(\frac{1}{2}\sigma^{2}y^{2}{)}^{-s}-\frac{2\tilde{c}s}{\sigma^{2}}{\left(\frac{\sigma^{2}}{2}\right)}^{-s}y^{\nu -2s-2}+O(y^{\nu -2Re(s)-2-\varepsilon }),y\to \infty ,$$

for *s* fixed, we see that the function

(5.15) $${\tilde{F}}_{1}(s):=\int_{y_{0}}^{\infty }\frac{1}{a+iy}\left(h{\left(y\right)}^{-s}-{\left(\frac{1}{2}\sigma^{2}y^{2}\right)}^{-s}\right)dy$$

is analytic for $-\tilde{\nu }<Re(s)<\frac{1}{2}$, and, clearly, for $0<Re(s)<\frac{1}{2}$ we have

(5.16) $$\tilde{F}(s)={\tilde{F}}_{1}(s)+{\tilde{G}}_{1}(s).$$

We have thus established the meromorphic continuation of $\tilde{F}$ to the strip $-\tilde{\nu }<Re(s)<\frac{1}{2}$. To continue $\tilde{F}$ even further, we look at the second term in Equation (5.14) and define

$$\begin{array}{rl} {\tilde{G}}_{2}(s): & =-\frac{2\tilde{c}s}{\sigma^{2}}{\left(\frac{\sigma^{2}}{2}\right)}^{-s}\int_{y_{0}}^{\infty }\frac{y^{\nu -2s-2}}{a+iy}dy\\ & =-\frac{2\tilde{c}\pi }{\sigma^{2}}{\left(\frac{\sigma^{2}}{2}\right)}^{-s}sa^{\nu -2s-2}\frac{e^{(2s-\nu +3)\pi i/2}}{\sin\pi (\nu -2s)}+\frac{2\tilde{c}s}{\sigma^{2}}{\left(\frac{\sigma^{2}}{2}\right)}^{-s}\int_{0}^{y_{0}}\frac{y^{\nu -2s-2}}{a+iy}dy\\ & =:G_{2}(s)+A_{2}(s) \end{array}$$

and the compensated function

$${\tilde{F}}_{2}(s):=\int_{y_{0}}^{\infty }\frac{1}{a+iy}\left(h{\left(y\right)}^{-s}-{\left(\frac{1}{2}\sigma^{2}y^{2}\right)}^{-s}+\frac{2\tilde{c}s}{\sigma^{2}}{\left(\frac{\sigma^{2}}{2}\right)}^{-s}y^{\nu -2s-2}\right)dy.$$

By Equation (5.14), the function ${\tilde{F}}_{2}$ is analytic for $Re(s)\in (-\tilde{\nu }-\varepsilon /2,(\nu -1)/2)$. Moreover, by definition we have

$${\tilde{F}}_{1}(s)={\tilde{F}}_{2}(s)+{\tilde{G}}_{2}(s),-\tilde{\nu }<Re(s)<\frac{\nu -1}{2},$$

and so the meromorphic continuation of $\tilde{F}$ to the region $-\tilde{\nu }-\varepsilon /2<Re(s)<\frac{1}{2}$ is established.

In order to shift the integration path in Equation (5.10) to the left, we have to ensure that the integral converges. This is the content of Lemma 7, which also yields the existence of an appropriate $y_{0}\geq 0$, to be used in the definition of $\tilde{F}$ in Equation (5.11). By the residue theorem, we obtain

(5.17) $$\begin{array}{rl} H(T)=\; & Res_{s=0}(MH)(s)T^{-s}+Res_{s=-\tilde{\nu }}(MH)(s)T^{-s}\\ & +\frac{1}{2\pi i}\int_{\kappa -i\infty }^{\kappa +i\infty }(MH)(s)T^{-s}ds,T\geq 0, \end{array}$$

where $\kappa =-\tilde{\nu }-\varepsilon /4$, and ℳ*H* now of course denotes the meromorphic continuation of the Mellin transform. We then compute the residues. According to Equations (5.11) and (5.16), the continuation of ℳ*H* in a neighborhood of *s* = 0 is given by $\Gamma (s)(A_{0}(s)+{\tilde{F}}_{1}(s)+{\tilde{G}}_{1}(s))$. Therefore,

(5.18) $$\begin{array}{rl} Res_{s=0}(MH)(s)T^{-s} & =A_{0}(0)+{\tilde{F}}_{1}(0)+A_{1}(0)+Res_{s=0}\Gamma (s)G_{1}(s)T^{-s}\\ & =Res_{s=0}\Gamma (s)G_{1}(s)T^{-s}\\ & =\frac{1}{2}\pi +i(\frac{1}{2}\gamma -\log(a\sigma /\sqrt{2})+\frac{1}{2}\log T), \end{array}$$

where *γ* is Euler’s constant. Note that $A_{0}(0)=-A_{1}(0)$ and ${\tilde{F}}_{1}(0)=0$ by definition. The remaining residue (Equation (5.18)) is straightforward to compute from Equation (5.13) (e.g., with a computer algebra system) and has real part $\frac{1}{2}\pi$. Notice that the logarithmic term log*T*, resulting from the *double* pole at zero (see the end of Section 4), appears only in the imaginary part. Recalling Equation (5.9), we see that the first term on the right-hand side of Equation (5.17) thus yields the first term of Equation (5.8).

Similarly, we compute for ν>1

$$\begin{array}{rl} Res_{s=-\tilde{\nu }}(MH)(s)T^{-s} & =Res_{s=-\tilde{\nu }}\Gamma (s)G_{2}(s)T^{-s}\\ & =\frac{\Gamma (-\tilde{\nu })}{2\pi }{\left[\frac{2\tilde{c}s}{\sigma^{2}}{\left(\frac{\sigma^{2}}{2}\right)}^{-s}\pi a^{\nu -2s-2}e^{(2s-\nu +3)\pi i/2}T^{-s}\right]}_{s=-\tilde{\nu }}. \end{array}$$

In the case ν=1, the function *G*_1_ also has a pole at $-\tilde{\nu }=-\frac{1}{2}$, and we obtain

$$\begin{array}{rl} Res_{s=-\tilde{\nu }}(MH)(s)T^{-s} & =Res_{s=-1/2}\Gamma (s)(G_{1}(s)+G_{2}(s))T^{-s}\\ & =\sqrt{\frac{\pi }{2}}\left(\frac{i\tilde{c}}{\sigma }-a\sigma \right)\sqrt{T}. \end{array}$$

A straightforward computation shows that the stated formula for $C_{\tilde{\nu }}$ is correct in both cases. The integral on the right-hand side of Equation (5.17) is clearly $O(T^{-\kappa })=o(T^{\tilde{\nu }})$, and so the proof is complete.

Lemma 7. *There is* $y_{0}\geq 0$ *such that the meromorphic continuation of* ℳ*H constructed in the proof of Theorem 5, which depends on y*_0_ *via the definition of* $\tilde{F}$ *in* *Equation (5.11)*, *decays exponentially as* |Im(s)|→∞.

Lemma 7 is proved in the Appendix.

## 6. Examples

We now apply our main results (Theorem 5 and Corollary 6) to several concrete models.

Example 8. *The NIG model has Laplace exponent*

$$\psi (z)=\frac{1}{2}\sigma^{2}z^{2}+\mu z+\delta \left(\sqrt{{\hat{\alpha }}^{2}-\beta^{2}}-\sqrt{{\hat{\alpha }}^{2}-{\left(\beta +z\right)}^{2}}\right),$$

*where* δ>0, $\hat{\alpha }>max\{\beta +1,-\beta \}$. (*The notation* $\hat{\alpha }$ *should avoid confusion with α from Theorem 1.) Since S is a martingale, we must have*

$$\mu =-\frac{1}{2}\sigma^{2}+\delta \left(\sqrt{{\hat{\alpha }}^{2}-{\left(\beta +1\right)}^{2}}-\sqrt{{\hat{\alpha }}^{2}-\beta^{2}}\right).$$

*The relation between μ and b from* *Equation (2.1)* *is* $\mu +\beta \delta /\sqrt{{\hat{\alpha }}^{2}-\beta^{2}}=b$, *as seen from the derivative of the Laplace exponent* ψ *at z = 0. The Lévy density is*

$$\frac{\nu (dx)}{dx}=\frac{\delta \hat{\alpha }}{\pi |x|}e^{\beta x}K_{1}(\hat{\alpha }|x|),$$

*where K*_1_ *is the modified Bessel function of second order and index 1.*

*First assume* σ=0. *Since* $K_{1}(x)∼1/x$ *for* x↓0, *condition* (H-α) *is satisfied with* α=1, *with* $c_{+}=c_{-}=\delta /\pi$. *The integrability condition in part (iv) of Theorem 1 is easily checked, and we conclude*

$$\underset{T↓0}{\lim}P[X_{T}\geq 0]=\frac{1}{2}+\frac{1}{\pi }\arctan(\frac{\mu }{\delta }),\sigma =0.$$

*Note that* $b^{*}=\mu =b-\frac{\delta \hat{\alpha }}{\pi }\int_{0}^{\infty }K_{1}(\hat{\alpha }x)(e^{\beta x}-e^{-\beta x})dx$. *By Lemma 2, the implied volatility slope of the NIG model thus satisfies*

$$\partial_{K}\sigma_{imp}(K,T){|}_{K=1}∼-\sqrt{2/\pi }\arctan(\mu /\delta )\cdot T^{-1/2},T↓0,\sigma =0,\mu \neq 0.$$

*Now assume that* σ>0. *Since* $\sqrt{{\hat{\alpha }}^{2}-{\left(\beta +z\right)}^{2}}=-iz+O(1)$ *as* Im(z)→∞,*Equation* (5.7) *becomes*

$$\psi (z)=\frac{1}{2}\sigma^{2}z^{2}+(\mu +i)z+O(1),Re(z)=a,Im(z)\to \infty .$$

*We can thus apply Theorem 5 to conclude that the ATM digital price satisfies*

$$P[X_{T}\geq 0]=\frac{1}{2}+\frac{\mu }{\sigma \sqrt{2\pi }}\sqrt{T}+o(\sqrt{T}),T↓0,\sigma >0.$$

*By part (i) of Corollary* 6, *the limit of the implied volatility slope is given by*

(6.1) $$\begin{array}{rl} & \underset{T↓0}{\lim}\partial_{K}\sigma_{imp}(K,T){|}_{K=1}=-\frac{\mu }{\sigma }-\frac{\sigma }{2}\\ & =\frac{\delta }{\sigma }(\sqrt{{\hat{\alpha }}^{2}-\beta^{2}}-\sqrt{{\hat{\alpha }}^{2}-{\left(\beta +1\right)}^{2}}),\sigma >0. \end{array}$$

This limit is positive if and only if $\beta >-\frac{1}{2}$.

**Figure 1.**
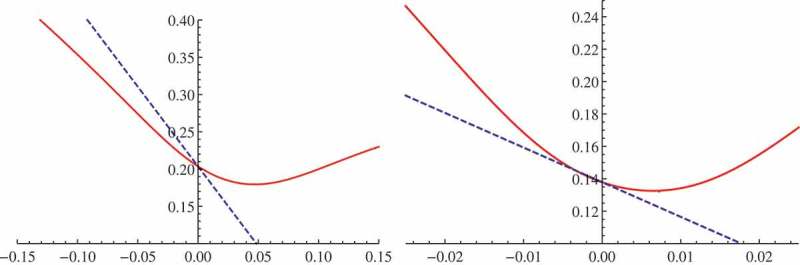
The volatility smile, as a function of log-strike, of the NIG model with parameters σ=0.085, $\hat{\alpha }=4.237$, β=−3.55, δ=0.167 and maturity T=0.1 (left panel), respectively, T=0.01 (right panel). The parameters were calibrated to S&P 500 call prices from Appendix A of Bu (2007). The dashed line is the slope approximation (Equation (6.1)). We did the calibration and the plots with Mathematica, using the Fourier representation of the call price.

See Figure 1 for a numerical example. Let us stress again that we identify the correct *sign* of the slope, while we find that explicit asymptotics do not approximate the *value* of the slope very accurately. Still, in the right panel of Figure 1, we have zoomed in at very short maturity to show that our approximation gives the asymptotically correct tangent in this example.

Example 9. *The Laplace exponent of the Meixner model is*

$$\psi (z)=\frac{1}{2}\sigma^{2}z^{2}+\mu z+2\hat{d}\log\frac{\cos(\hat{b}/2)}{\cosh\frac{1}{2}(-\hat{a}iz-i\hat{b})},$$

*where* $\hat{d}>0$, $\hat{b}\in (-\pi ,\pi )$, and $0<\hat{a}<\pi -\hat{b}$. (*We follow the notation of* Schoutens 2002, *except that we write μ instead of m, and* $\hat{a},\hat{b},\hat{d}$ *instead of a,b,d*.) *The Lévy density is*

$$\frac{\nu (dx)}{dx}=\hat{d}\frac{\exp(\hat{b}x/\hat{a})}{x\sin h(\pi x/\hat{a})}.$$

*We can proceed analogously to Example 8. For* σ=0, *we again apply part (iv) of Theorem 1, with* α=1, *where now* $c_{+}=c_{-}=\hat{d}\hat{a}/\pi$. *Consequently*,

$$\underset{T↓0}{\lim}P[X_{T}\geq 0]=\frac{1}{2}+\frac{1}{\pi }\arctan\left(\frac{\mu }{\hat{a}\hat{d}}\right),\sigma =0,$$

and

$$\partial_{K}\sigma_{imp}(K,T){|}_{K=1}∼-\sqrt{2/\pi }\arctan\left(\frac{\mu }{\hat{a}\hat{d}}\right)\cdot T^{-1/2},T↓0,\sigma =0,\mu \;\neq \;0.$$

Now assume σ>0. The expansion of the Laplace exponent is

$$\psi (z)=\frac{1}{2}\sigma^{2}z^{2}+(\mu +\hat{a}\hat{d}i)z+O(1),Re(z)=a,Im(z)\to \infty .$$

By Theorem 5, the ATM digital price in the Meixner model thus satisfies

$$P[X_{T}\geq 0]=\frac{1}{2}+\frac{\mu }{\sigma \sqrt{2\pi }}\sqrt{T}+o(\sqrt{T}),T↓0.$$

The limit of the implied volatility slope is given by

$$\underset{T↓0}{\lim}\partial_{K}\sigma_{\text{imp}}(K,T){|}_{K=1}=-\frac{\mu }{\sigma }-\frac{\sigma }{2}=\frac{2\hat{d}}{\sigma }\log\left(\frac{\cos(\hat{b}/2)}{\cosh\frac{1}{2}(-(\hat{a}+\hat{b})i)}\right),\sigma >0.$$

Example 10. *The Laplace exponent of the CGMY model is*

(6.2) $$\psi (z)=\frac{1}{2}\sigma^{2}z^{2}+\mu z+C\Gamma (-Y)((M-z{)}^{Y}-M^{Y}+(G+z{)}^{Y}-G^{Y}),$$

*where we assume* C>0, G>0, M>1, 0<Y<2,*and* Y≠1.

*The case* σ=0 *and* Y∈(0,1) *need not be discussed, as it is a special case of Proposition 8.5 in* Andersen and Lipton (2013). *Our Proposition* 3 *could also be applied, as the CGMY process has finite variation in this case.*

*If* σ=0 *and* Y∈(1,2), *then the ATM digital call price converges to* $\frac{1}{2}$, *and the slope explodes, of order* $T^{1/2-1/Y}$. *This is a special case of Corollary 3.3 in* Figueroa-López and Ólafsson (2015). *Note that Proposition 8.5 in* Andersen and Lipton (2013) *is not applicable here, because the constant* $C_{M}$ *from this proposition vanishes for the CGMY model, and so the leading term of the slope is not obtained. Theorem* 1 *(iv) from our* *Section 2* *is not useful, either; it gives the correct digital call limit price* $\frac{1}{2}$, *but does not provide the second-order term necessary to get slope asymptotics.*

*We now proceed to the case* σ>0, *which is our main focus. The expansion of* ψ *at i∞ is*

$$\psi (z)=\frac{1}{2}\sigma^{2}z^{2}+c_{Y}z^{Y}+\mu z+O(z^{Y-1}),Re(z)=a,Im(z)\to \infty ,$$

with the complex constant $c_{Y}:=C\Gamma (-Y)(1+e^{-i\pi Y})$. First assume 0<Y<1. Then we proceed analogously to the preceding examples, applying Theorem 5 and Corollary 6. The ATM digital price thus satisfies

(6.3) $$P[X_{T}\geq 0]=\frac{1}{2}+\frac{\mu }{\sigma \sqrt{2\pi }}\sqrt{T}+o(\sqrt{T}),T↓0,$$

and the limit of the implied volatility slope is given by

(6.4) $$\begin{array}{r} \underset{T↓0}{\lim}\partial_{K}\sigma_{imp}(K,T){|}_{K=1}=-\frac{\mu }{\sigma }-\frac{\sigma }{2}=\frac{1}{\sigma }C\Gamma (-Y)((M-1{)}^{Y}-M^{Y}+(G+1{)}^{Y}-G^{Y}). \end{array}$$

*Now assume* 1<Y<2. *In principle, Theorem 5 is applicable, with* ν=Y *; however, the constant* $C_{\tilde{\nu }}$ *in* *Equation (5.8)* *is zero, and so we do not get the second term of the expansion immediately. What happens is that the Mellin transform of H (see the proof of Theorem 5) may have further poles in* $-\frac{1}{2}<Re(s)<0$, *but none of them gives a contribution, since the corresponding residues have zero real part. Therefore*, *Equations* (6.3) *and* (6.4) *are true also for* 1<Y<2. *See Pinter’s (in preparation*) *PhD thesis for details. Note that* *Equations* (6.3) *and* (6.4) *also follow from concurrent work by* Figueroa-López and Ólafsson (2015). *For* 0<Y<1, *they also follow from Proposition 8.5 in* Andersen and Lipton (2013), *but not for* 1<Y<2, *because then the constant* $C_{M}$ *from that proposition vanishes when specializing it to the CGMY model.*

In the following example, we discuss the generalized tempered stable model. The tempered stable model, which is investigated in Andersen and Lipton (2013), is obtained by setting $\alpha_{-}=\alpha_{+}$.

Example 11. *The generalized tempered stable process* (Cont and Tankov 2004) *is a generalization of the CGMY model, with Lévy density*

$$\frac{\nu (dx)}{dx}=\frac{C_{-}}{|x{|}^{1+\alpha_{-}}}e^{-\lambda_{-}|x|}1_{\left(-\infty ,0\right)}(x)+\frac{C_{+}}{|x{|}^{1+\alpha_{+}}}e^{-\lambda_{+}|x|}1_{\left(0,\infty \right)}(x),$$

*where* $\alpha_{\pm }<2$ *and* $C_{\pm },\lambda_{\pm }>0$. *For* $\alpha_{\pm }\notin \{0,1\}$ *the Laplace exponent of the generalized tempered stable process is*

$$\begin{array}{rl} \psi (z)=\; & \frac{1}{2}\sigma^{2}z^{2}+\mu z+\Gamma (-\alpha_{+})C_{+}((\lambda_{+}-z{)}^{\alpha_{+}}-\lambda_{+}^{\alpha_{+}})\\ & +\Gamma (-\alpha_{-})C_{-}((\lambda_{-}+z{)}^{\alpha_{-}}-\lambda_{-}^{\alpha_{-}}). \end{array}$$

*For* σ>0, $\alpha_{+}\in (1,2)$, *and* $\alpha_{-}<\alpha_{+}$ *we have the following expansion*:

$$\psi (z)=\frac{1}{2}\sigma^{2}z^{2}+\Gamma (-\alpha_{+})C_{+}e^{-i\pi \alpha_{+}}z^{\alpha_{+}}+O(z^{\max\{1,\alpha_{-}\}}),Re(z)=a,Im(z)\to \infty .$$

*We now apply Theorem 5 with* $\nu =\alpha_{+}$, *and find that the second order expansion of the ATM digital call is*

$$P[X_{T}\geq 0]=\frac{1}{2}+C_{\tilde{\nu }}T^{\tilde{\nu }}+o(T^{\tilde{\nu }}),T↓0,$$

*with* $\tilde{\nu }=1-\alpha_{+}/2\in (0,\frac{1}{2})$ *and the real constant*

$$C_{\tilde{\nu }}=\frac{\tilde{\nu }}{2\pi }{\left(\frac{1}{2}\sigma^{2}\right)}^{\tilde{\nu }-1}\Gamma (-\alpha_{+})C_{+}\underset{=\sin(-\pi (1+\alpha_{+}/2))}{\underbrace{Im(e^{-i\pi \tilde{\nu }}e^{-i\pi \alpha_{+}})}}\Gamma (-\tilde{\nu }).$$

*By Corollary 6 (ii), the ATM implied volatility slope explodes, but slower than T^–1/2^*:

$$\partial_{K}\sigma_{imp}(K,T){|}_{K=1}∼-\sqrt{2\pi }C_{\tilde{\nu }}T^{\tilde{\nu }-1/2},T↓0.$$

*Note that these results also follow from the concurrent paper (*Figueroa-López and Ólafsson 2015 *), which treats tempered stable-like models.*

*If* σ>0 *and* $\alpha_{+}<1$, *then part (i) of Corollary 6 is applicable, and formulas analogous to* *Equations (6.3)* *and* *(6.4)* *hold.*

## 7. Robustness of Lee’s Moment Formula

As we have already mentioned, our first-order slope approximations give limited accuracy for the size of the slope, but usually succeed at identifying its sign, i.e., whether the smile increases or decreases at the money. It is a natural question whether this sign gives information on the smile as a whole: If the slope is positive, does it follow that the right wing is steeper than the left one, and vice versa? To deal with this issue, recall Lee’s moment formula (Lee 2004a). Under the assumption that the critical moments *z*_+_ and *z*-, defined in Equations (5.1) and (5.2), are finite, Lee’s formula states that

(7.1) $$\underset{k\to \infty }{\lim\;sup}\frac{\sigma_{imp}(K,T)}{\sqrt{k}}=\sqrt{\frac{\Psi (z_{+}-1)}{T}}$$

and

(7.2) $$\underset{k\to -\infty }{\lim\;sup}\frac{\sigma_{imp}(K,T)}{\sqrt{-k}}=\sqrt{\frac{\Psi (-z_{-})}{T}},$$

where T>0 is fixed, k=logK and $\Psi (x):=2-4(\sqrt{x^{2}+x}-x)$. According to Lee’s formula, the slopes of the wings depend on the size of the critical moments. In Lévy models, the critical moments do not depend on *T*. The compatibility property we seek now becomes

(7.3) $$\underset{k\to \infty }{\lim}\frac{\sigma_{imp}(K,T)}{\sqrt{k}}>\underset{k\to -\infty }{\lim}\frac{\sigma_{imp}(K,T)}{\sqrt{-k}}for\;allT>0$$

if and only if

(7.4) $$\partial_{K}\sigma_{imp}(K,T){|}_{K=1}>0for\;all\;sufficiently\;small\;T.$$

That is, the right wing of the smile is steeper than the left wing deep *out-of-the-money* if and only if the small-maturity *ATM* slope is positive. We now show that this is true for several infinite activity Lévy models. By our methods, this can certainly be extended to other infinite activity models. It does not hold, though, for the Merton and Kou jump diffusion models. The parameter ranges in the following theorem are the same as in the examples in Section 6.

Theorem 12. *Equations* (7.3) *and* (7.4) *are equivalent for the following models. For the latter three, we assume that* σ>0 *or* μ ≠ 0.

- *Variance gamma with* σ=0, $b_{0}\;\neq \;0$
- *NIG*
- *Meixner*
- *CGMY*

Put differently, these models are *not* capable (at short maturity) of producing a smile that has, say, its minimum to the left of logK=k=0, and thus a positive ATM slope, but whose left wing is steeper than the right one.

*Proof*. The critical moments are clearly finite for all of these models. Moreover, it is well known that the limsup in Equations (7.1) and (7.2) can typically be replaced by a genuine limit, for instance, using the criteria given by Benaim and Friz (2008). Their conditions on the mgf are easily verified for all our models; in fact Benaim and Friz (2008) explicitly treat the variance gamma model with $b_{0}=0$ and the NIG model. We thus have to show that Equation (7.4) is equivalent to $\Psi (z_{+}-1)>\Psi (-z_{-})$. Since Ψ is strictly decreasing on (0,∞), the latter condition is equivalent to $z_{+}-1<-z_{-}$. It remains to check the equivalence

(7.5) $$z_{+}-1<-z_{-}\Leftrightarrow (7.4).$$

The mgf of the variance gamma model is (see Madan, Carr, and Chang 1998)

$$M(z,T)=e^{Tb_{0}z}(1-\theta \nu z-\frac{1}{2}{\hat{\sigma }}^{2}\nu z^{2}{)}^{-T/\nu },$$

where $\hat{\sigma },\nu >0$ and θ∈R. Its paths have finite variation, and so Proposition 3 shows that Equation (7.4) is equivalent to $b_{0}<0$. The critical moments are

$$z_{\pm }=-\frac{\nu \theta \pm \sqrt{2\nu {\hat{\sigma }}^{2}+\nu^{2}\theta^{2}}}{\nu {\hat{\sigma }}^{2}},$$

and we have $-z_{-}+1-z_{+}=1+2\theta /{\hat{\sigma }}^{2}$. This is positive if and only if

$$b_{0}=\nu^{-1}\log(1-\theta \nu -\frac{1}{2}{\hat{\sigma }}^{2}\nu )<0,$$

which yields Equation (7.5).

As for the other three models, first suppose that σ>0. The examples in Section 6 show that Equation (7.4) is equivalent to $\mu <-\frac{1}{2}\sigma^{2}$. The critical moments of the NIG model are $z_{+}=\hat{\alpha }-\beta$ and $z_{-}=-\hat{\alpha }-\beta$. Therefore, $z_{+}-1<-z_{-}$ if and only if $\beta >-\frac{1}{2}$, and this is indeed equivalent to

$$\mu +\frac{1}{2}\sigma^{2}=\delta (\sqrt{{\hat{\alpha }}^{2}-{\left(\beta +1\right)}^{2}}-\sqrt{{\hat{\alpha }}^{2}-\beta^{2}})<0.$$

For the Meixner model, we have $z_{\pm }=(\pm \pi -\hat{b})/\hat{a}$, which yields $-z_{-}+1-z_{+}=1+2\hat{b}/\hat{a}$. On the other hand,

$$\mu +\frac{1}{2}\sigma^{2}=-2\hat{d}\log\frac{\cos(\hat{b}/2)}{\cos((\hat{a}+\hat{b})/2)},$$

which is negative if and only if $\cos(\hat{b}/2)>\cos((\hat{a}+\hat{b})/2)$, and this is equivalent to $\hat{a}+2\hat{b}>0$.

Finally, in case of the CGMY model, we have

$$\mu +\frac{1}{2}\sigma^{2}=-C\Gamma (-Y)((M-1{)}^{Y}-M^{Y}+(G+1{)}^{Y}-G^{Y}).$$

Since, for Y∈(0,1), Γ(−Y)<0 and the function $x\mapsto x^{Y}-(x+1{)}^{Y}$ is strictly increasing on (0,∞), we see that $\mu +\frac{1}{2}\sigma^{2}<0$ if and only if M−1<G. This is the desired condition, since the explicit Equation (6.2) shows that $z_{+}=M$ and $z_{-}=-G$. The case Y∈(1,2) is analogous.

It remains to treat the case σ=0. First, note that the critical moments do not depend on *σ*. Furthermore, from the examples in Section 6, we see that Equation (7.4) holds if and only if μ<0. Now observe that adding a Brownian motion $\sigma W_{t}$ to a Lévy model adds $-\frac{1}{2}\sigma^{2}$ to the drift, if the martingale property is to be preserved. Therefore, the assertion follows from what we have already proved about σ>0.

## 8. Conclusion

Our main result (Corollary 6) translates asymptotics of the log-underlying’s mgf to first-order asymptotics for the ATM implied volatility slope. Checking the requirements of Corollary 6 only requires Taylor expansion of the mgf, which has an explicit expression in all models of practical interest. Higher-order expansions can be obtained by the same proof technique, if desired. They will follow in a relatively straightforward way from higher-order expansions of the mgf, by collecting further residues of the Mellin transform. In future work, we hope to connect our assumptions on the mgf with properties of the Lévy triplet, which should give additional insight on how the slope depends on model characteristics.

## Appendix Proofs of lemmas 4 and 7

*Proof of Lemma* 4. Since $S=e^{X}$ is a martingale, we have $\psi^{\prime }(0)=E[X_{1}]<0$. Then ψ(0)=0 implies that ψ(a)<0 for all sufficiently small a>0. In fact, it easily follows from ψ(1)=0 and the concavity of *Ψ* that all a∈(0,1) satisfy ψ(a)<0. Let us fix such an *a*. From

$$Re(-\psi (a+iy))=-\psi (a)+\frac{1}{2}\sigma^{2}y^{2}+\int_{R}e^{ax}\underset{\geq 0}{\underbrace{\left(1-\cos(yx)\right)}}\nu (dx)$$

we obtain that the function h(y):=−ψ(a+iy), y≥0, satisfies

(A.1) $$Reh(y)>\frac{1}{2}\sigma^{2}y^{2}\geq 0,y\geq 0.$$

For $0<Re(s)<\frac{1}{2}$, define the function

$$g(T)=T^{Re(s)-1}\int_{0}^{\infty }\frac{e^{-TRe(h(y))}}{|a+iy|}dy,T>0.$$

Using Fubini’s theorem and substituting TRe(h(y))=u, we then calculate for Re(s)>0

$$\int_{0}^{\infty }g(T)\text{d}T=\int_{0}^{\infty }\frac{1}{\left|a+iy\right|}\int_{0}^{\infty }e^{-TRe(h(y))}T^{Re\;(s)-1}\text{d}T\text{d}y$$

$$=\int_{0}^{\infty }\frac{Re{\left(h(y)\right)}^{-Re(s)}}{|a+iy|}\left(\int_{0}^{\infty }e^{-u}u^{Re(s)-1}du\right)dy$$

$$=\Gamma (Re(s))\int_{0}^{\infty }\frac{Re{\left(h(y)\right)}^{-Re(s)}}{|a+iy|}dy.$$

From (A.1), we get

$$\int_{0}^{\infty }\frac{Re{\left(h(y)\right)}^{-Re(s)}}{|a+iy|}dy\leq (\frac{1}{2}\sigma^{2}{)}^{-Re(s)}\int_{0}^{\infty }\frac{y^{-2Re(s)}}{|a+iy|}dy.$$

The restriction $Re(s)<\frac{1}{2}$ ensures that the last integral is finite and thus the integrability of *g*. Using the dominated convergence theorem and Fubini’s theorem, the Mellin transform of *H* can now be calculated as

$$\int_{0}^{\infty }H(T)T^{s-1}dT=\int_{0}^{\infty }\frac{1}{a+iy}\int_{0}^{\infty }e^{-Th(y)}T^{s-1}dTdy.$$

The substitution Th(y)=u gives us the result. Note that *h*(*y*) is in general non-real; it is easy to see, though, that Euler’s integral

$$\Gamma (s)=\int_{0}^{\infty }u^{s-1}e^{-u}du,Re(s)>0,$$

still represents the gamma function if the integration is performed along any complex ray emanating from zero, as long as the ray stays in the right half-plane. The latter holds, since Re(h(y))>0.

It remains to prove the exponential decay of the Mellin transform MH(s)=Γ(s)F(s) for large |Im(s)|. First, note that

$$Im\psi (a+iy)=by+\sigma^{2}ay+\int_{R}(e^{ax}\sin xy+xy)\nu (dx)$$

=O(y),y→∞,

which together with Equation (A.1) yields the existence of an ε>0 such that $|\arg h(y)|\leq \frac{1}{2}\pi -\varepsilon$ for all y≥0. We then estimate, with $Re(s)\in (0,\frac{1}{2})$ fixed,

$$|F(s)|\leq \int_{0}^{\infty }\frac{e^{-Re(s\log h(y))}}{|a+iy|}dy$$

$$=\int_{0}^{\infty }\frac{e^{-Re(s)\log|h(y)|+Im(s)\arg h(y)}}{|a+iy|}dy$$

$$\leq e^{(\pi /2-\varepsilon )|Im(s)|}\int_{0}^{\infty }\frac{{\left(\frac{1}{2}\sigma^{2}y^{2}\right)}^{-Re(s)}}{|a+iy|}dy.$$

The integral converges, and thus this estimate is good enough, since Stirling’s formula yields $|\Gamma (s)|=\exp(-\frac{1}{2}\pi |Im(s)|(1+o(1)))$.

*Proof of Lemma* 7. Recall that, in the proof of Theorem 5, we defined the following meromorphic continuation of *F*(*s*), to the strip $-\tilde{\nu }-\frac{1}{2}\varepsilon <Re(s)<\frac{1}{2}$:

$$A_{0}(s)+{\tilde{G}}_{1}(s)+{\tilde{F}}_{1}(s),-\tilde{\nu }<Re(s)<\frac{1}{2},$$

$$A_{0}(s)+{\tilde{G}}_{1}(s)+{\tilde{G}}_{2}(s)+{\tilde{F}}_{2}(s),-\tilde{\nu }-\frac{1}{2}\varepsilon <Re(s)<\frac{1}{2}(\nu -1).$$

As noted at the end of the proof of Lemma 4, Stirling’s formula implies $|\Gamma (s)|=\exp(-\frac{1}{2}\pi |Im(s)|(1+o(1)))$. By Equation (5.5), it thus suffices to argue that the continuation of F(s) is $O(\exp((\frac{1}{2}\pi -\varepsilon )|Im(s)|))$ for some ε>0. The functions ${\tilde{G}}_{1}$ and ${\tilde{G}}_{2}$ are clearly O(1). As for $A_{0}$, defined in Equation (5.11), we have

$$|A_{0}(s)|\leq \int_{0}^{y_{0}}\frac{e^{-Re(s\log h(y))}}{|a+iy|}dy$$

$$=\int_{0}^{y_{0}}\frac{|h(y{)|}^{-Re(s)}e^{Im(s)\arg h(y)}}{|a+iy|}dy.$$

Now note that

$$|h(y){|}^{-Re(s)}\leq (\begin{array}{cc} {\left(\frac{1}{2}\sigma^{2}y^{2}\right)}^{-Re(s)} & 0<Re(s)<\frac{1}{2},\\ {\left(\max_{0\leq y\leq y_{0}}|h(y)|\right)}^{-Re(s)} & Re(s)\leq 0, \end{array}$$

and that

$$\exp(Im(s)\arg h(y))\leq \exp((\frac{\pi }{2}-\varepsilon )|Im(s)|)$$

for some ε>0, as argued in the proof of Lemma 4.

It remains to establish a bound for ${\tilde{F}}_{1}$, defined in Equation (5.15). (The bound for ${\tilde{F}}_{2}$ is completely analogous, and we omit the details.) In what follows, we assume that $-\tilde{\nu }<Re(s)<\frac{1}{2}$. By Equation (5.12), we have (where the *O* is uniform w.r.t. *s*, and $y_{0}\geq 0$ is still arbitrary):

$${\tilde{F}}_{1}(s)=\int_{y_{0}}^{\infty }\frac{1}{a+iy}\left({\left(\frac{1}{2}\sigma^{2}y^{2}\right)}^{-s}{\left(1+O(y^{\nu -2})\right)}^{-s}-{\left(\frac{1}{2}\sigma^{2}y^{2}\right)}^{-s}\right)dy$$

(A.2) $$=\int_{y_{0}}^{\infty }\frac{1}{a+iy}(\frac{1}{2}\sigma^{2}y^{2}{)}^{-s}\left({\left(1+O(y^{\nu -2})\right)}^{-s}-1\right)dy.$$

We now choose *y*_0_ such that, for some constant $C_{0}>0$,

$$|\log|1+O(y^{\nu -2})||\leq \frac{1}{4}\pi ,$$

$$|arg(1+O(y^{\nu -2}))|\leq \frac{1}{4}\pi ,$$

$$|\log(1+O(y^{\nu -2}))|\leq C_{0}y^{\nu -2},$$

hold for all $y\geq y_{0}$. (By a slight abuse of notation, here $O(y^{\nu -2})$ of course denotes the function hiding behind the $O(y^{\nu -2})$ in Equation (A.2).) For all w∈C, we have the estimate

$$|e^{w}-1|\leq |w|e^{|Re(w)|}.$$

Using this in Equation (A.2), we find

$$\left|{\left(1+O(y^{\nu -2})\right)}^{-s}-1\right|=\left|\exp(-s\log(1+O(y^{\nu -2})))-1\right|$$

$$\leq |s\log(1+O(y^{\nu -2}))|\cdot \exp(|Re(s\log(1+O(y^{\nu -2}))|)$$

$$\leq C_{1}|s|y^{\nu -2}\exp(\frac{1}{4}\pi |Im(s)|),$$

where $C_{1}=C_{0}\exp(\frac{1}{4}\pi \sup_{s}|Re(s)|)$, and thus

$$|{\tilde{F}}_{1}(s)|\leq C_{2}|s|e^{\frac{1}{4}\pi |Im(s)|}\int_{y_{0}}^{\infty }y^{-2Re(s)+\nu -3}dy$$

$$=\exp(\frac{1}{4}\pi |Im(s)|(1+o(1))).$$
